# Soft X-ray Atmospheric Pressure Photoionization in Liquid Chromatography–Mass Spectrometry

**DOI:** 10.1021/acs.analchem.1c01127

**Published:** 2021-07-01

**Authors:** Juha-Pekka Hieta, Roope Vesander, Mikko Sipilä, Nina Sarnela, Risto Kostiainen

**Affiliations:** † Drug Research Program and Division of Pharmaceutical Chemistry and Technology, Faculty of Pharmacy, University of Helsinki, P.O. Box 56, FI-00014 Helsinki, Finland; ‡ Institute for Atmospheric and Earth System Research/Physics, Faculty of Science, University of Helsinki, P.O. Box 64, FI-00014 Helsinki, Finland

## Abstract

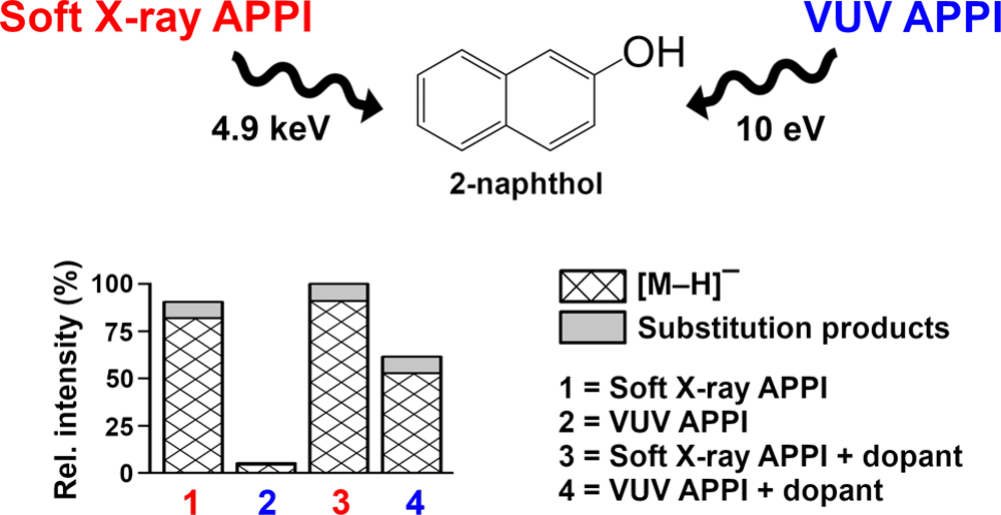

Soft X-ray atmospheric pressure photoionization (soft X-ray APPI) as an ionization method in liquid chromatography–mass spectrometry (LC-MS) is presented. The ionization mechanism was examined with selected test compounds in the negative ion mode, using soft X-ray APPI source emitting 4.9 keV photons. Test compounds with an acidic group were ionized by a proton transfer reaction, producing deprotonated molecules ([M – H]^−^), whereas compounds having positive electron affinity were ionized by a charge exchange reaction, producing negative molecular ions (M^–•^). Soft X-ray APPI does not require a dopant to achieve high ionization efficiency, which is an advantage compared with vacuum ultraviolet APPI with 10 eV photons, in which a dopant is needed to improve ionization efficiency. The energy of the soft X-ray photons is in the keV range, which is high enough to displace a valence electron and often also inner shell electrons from LC eluents and atmospheric gases, initiating an efficient ionization process in the negative ion mode.

The most common atmospheric pressure ion sources in liquid chromatography–mass spectrometry (LC-MS) are electrospray ionization (ESI), atmospheric pressure chemical ionization (APCI), and atmospheric pressure photoionization (APPI). APPI can efficiently ionize a wide range of compounds with different polarities and plays a special role in the analysis of nonpolar neutral compounds, such as steroids, polyaromatic hydrocarbons, and terpenes, which may be poorly ionized with ESI or APCI.^1,2^ In APPI, the ionization is initiated with 10 eV photons emitted by a vacuum ultraviolet (VUV) lamp. Since the ionization energies (IE) of most used LC eluents (e.g., methanol and acetonitrile) are higher than 10 eV, they are not efficiently ionized by VUV APPI. Therefore, a dopant having an IE below 10 eV, such as toluene, chlorobenzene, or anisole, is commonly added to the eluent to enhance the ionization efficiency. In the ionization process,^3^ the dopant is first photoionized by 10 eV photons, and a dopant radical cation is formed. If the IE of the analyte is smaller than that of the dopant, charge exchange reaction may occur, and a radical cation of the analyte is formed. In the other ionization pathway, the dopant donates a proton to the eluent molecule, which may react with the analyte by a proton transfer reaction if the proton affinity of the analyte is higher than that of the solvent molecule or its cluster. In the negative ion mode, the photoionization of the dopant forms thermal electrons that initiate the reactions leading to the ionization of analytes. The compounds with high electron affinity (EA) are ionized by electron capture or by charge exchange, and the compounds with high gas-phase acidity are ionized by proton transfer.^4^ The drawback of VUV APPI is that the use of dopant complicates the method, and commonly used dopants such as toluene and chlorobenzene are harmful for environment and health.

Higher-energy photons, such as soft X-ray photons, can efficiently ionize atoms and molecules without using a dopant. In the soft X-ray regime, photons have an energy of about 0.1–10 keV, which is about 10–1000 times higher than the energy of VUV photons. The energy of the soft X-ray photons is high enough to displace not only a valence electron but often also inner shell electrons, producing single and multiple charged compounds.^5−7^ The soft X-ray photons generated by a synchrotron light source or by an X-ray tube have been used to study the ionization and fragmentation of different types of small molecules, such as vanillin,^6^ alcohols,^8,9^ hydrocarbons,^5,7,10^ and amino acids.^11^ In all these studies, the compounds were ionized in the vacuum of a mass spectrometer, while few MS studies are available, in which soft X-ray photons were used for the ionization of compounds at atmospheric pressure. Riebe et al. examined the formation of reactant ions at atmospheric pressure from different gases and gas mixtures and ionization of alkyl nitrates in the negative ion mode, using 2.8 keV photons produced by a miniaturized soft X-ray APPI source.^12^ The same soft X-ray source was applied in the positive ion atmospheric pressure chemical ionization (APCI) of volatile organic compounds produced by various fungi, using gas chromatography-MS.^13^ Soft X-ray ion source has also been applied in different APCI methods aiming for selective ionization of atmospheric vapors, such as sulfuric acid, low-volatility organic compounds, and amines, in the negative ion mode.^14−16^ Thus far, soft X-ray ionization has not been presented as an ionization method in LC-MS.

Here, we present soft X-ray APPI as an ionization method in LC-MS for the first time. The ionization mechanism is examined with selected test compounds having different gas-phase energetics in the negative ion mode, using soft X-ray APPI source emitting 4.9 keV photons. The ionization efficiency of soft X-ray APPI is compared with that of VUV APPI in negative ion mode, and the feasibility of soft X-ray APPI is demonstrated in the LC-MS analysis of the selected test compounds.

## Experimental Section

**Figure 1:**
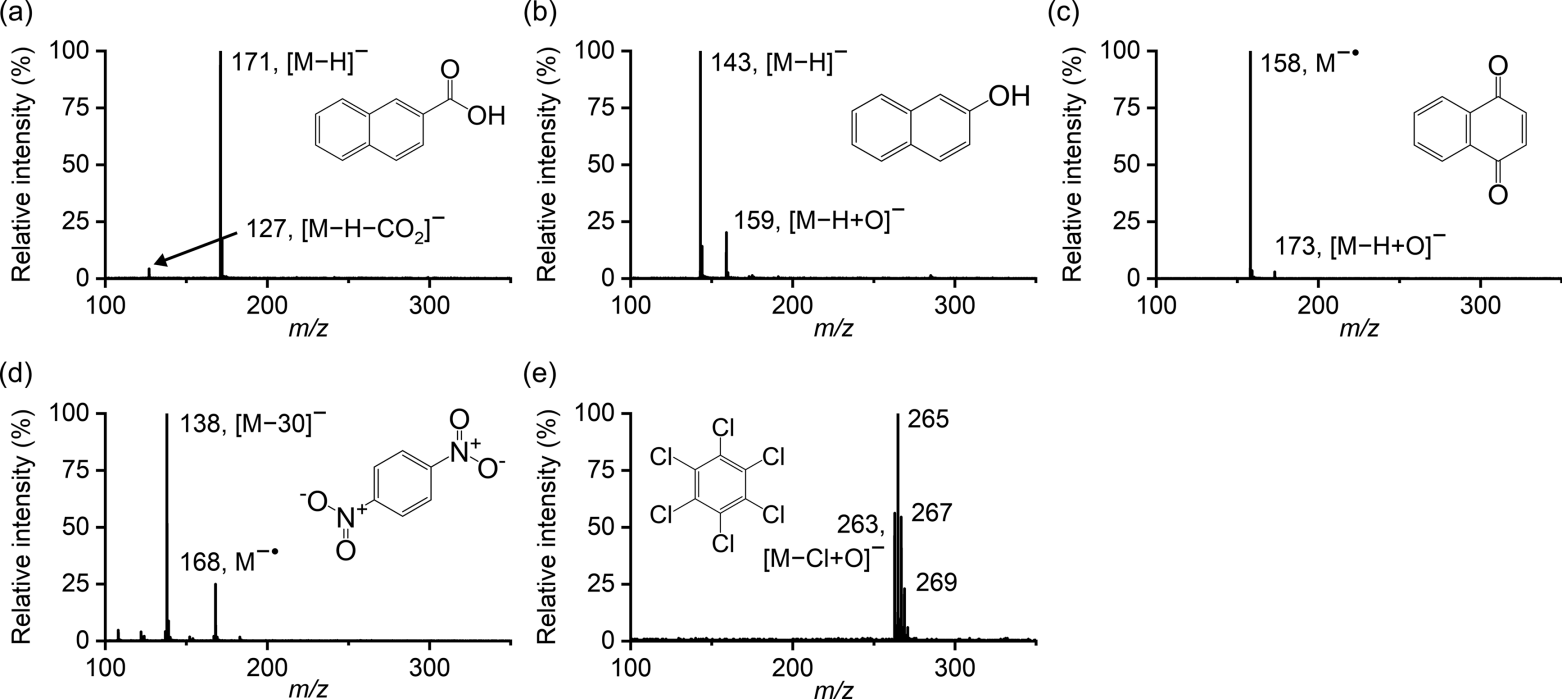
Background subtracted atmospheric pressure soft X-ray photoionization mass spectra of the test compounds: (a) 2-naphthoic acid, (b) 2-naphthol, (c) 1,2-naphthoquinone, (d) 1,4-dinitrobenzene, and (e) hexachlorobenzene. The spectra were recorded from the LC-MS run.

The selected test compounds, 2-naphthoic acid, 2-naphthol, 1,4-naphthoquinone, 1,4-dinitrobenzene, and hexachlorobenzene (Figure 1), were purchased from Sigma-Aldrich (Steinheim, Germany and Schweiz, Switzerland). Acetonitrile, methanol, and toluene were purchased from Honeywell International (Seelze, Germany). All chemicals were of analytical or chromatographic grade. The water was purified in a Milli-Q water purification system (Millipore, Molsheim, France). All test compounds were dissolved in a mixture of acetonitrile/water (50/50) to create a 100 ng mL^–1^ standard solution mixture for the mass spectra measurements. A standard solution mixture of 10 μg mL^–1^ was prepared in acetonitrile/water (90/10) with 0.1% formic acid for the LC-MS measurements.

All samples were analyzed with a Xevo quadrupole time-of-flight mass spectrometer (Q-TOF-MS) (Waters Corp., Manchester, UK) instrument that was coupled with an ACQUITY UPLC (Waters Corp., Milford, MA, USA). In the mass spectra measurements, the integrated fluidics system of the MS was used to deliver the sample directly to the ion source at a flow rate of 90 μL min^–1^. In the LC-MS experiments, an Acquity ultraperformance liquid chromatographic ethylene-bridged hybrid (UPLC BEH) C-18 column (100 mm × 2.1 mm i.d., 1.7 μm particle size) was used for chromatographic separation of the test compounds. Eluent A was 5% acetonitrile in Milli-Q water, and eluent B was 100% acetonitrile. The gradient was as follows: 10% B for 0–2 min, 50% B for 2–4 min, 100% B for 4–5.1 min, and 10% B for 5.1–8.0 min. The flow rate of the mobile phase was 400 μL min^–1^, the injection volume was 3 μL with partial loop with needle-overfill injection mode, and the column temperature was 40 °C. In all dopant-assisted measurements, toluene was infused with a syringe pump (Pump 11 Elite, Harvard apparatus; Harvard Bioscience Inc., Holliston, MA, USA) to the solvent line before the ion source to create a 10% dopant solution.

The soft X-ray and VUV APPI ion sources were built in-house to the frame of the commercial Waters Zspray Nanoflow ion source. The plastic enclosure of the Nanoflow metal frame was removed, and the nano-ESI probe was replaced with an APCI nebulizer from the Zspray APPI/APCI ion source and positioned to the same distance from the MS inlet as in the APPI/APCI source. The Nanoflow frame was more open than the APPI/APCI source and had built-in XY stages, allowing a separate soft X-ray source (PhotoIonBar L12536; Hamamatsu Photonics K.K., Hamamatsu, Japan) or a krypton discharge VUV lamp (PKR 100; Heraeus Noblelight Ltd., Cambridge, UK) to be attached and placed near the MS inlet region. The VUV lamp emitted 10.0 and 10.6 eV photons. The photon energy in the soft X-ray source was 4.9 keV, and the source was powered with a separate controller (C12537; Hamamatsu). Only one of the emitters was directed toward the MS inlet at a time. The VUV APPI and soft X-ray emitters were positioned at the same distance from the MS inlet. Aluminum foil was wrapped around the ion source frame to block the harmful soft X-ray photons, and the minimum working distance from the soft X-ray source was 2 m when the source was on to ensure safe working conditions.

The mass spectra were acquired in the mass range of mass-to-charge ratio (*m*/*z*) 30–500 with a data acquisition frequency of 2 Hz. The cone and desolvation gas flow rates were 0 and 800 L h^–1^, and the source and probe temperatures were 100 and 200 °C, respectively. In the LC-MS experiments, the probe temperature was set to 600 °C, due to the increased solution flow rate. The extraction and sampling cone values were set to 4.0 and 30.0, respectively.

## Results and Discussion

The ionization process in negative ion soft X-ray APPI-MS was examined with the selected test compounds (Figure 1), including acidic groups (2-naphthol and 2-naphthoic acid) and those having positive EAs (1,4-naphthoquinone, 1,4-dinitrobenzene, and hexachlorobenzene). The same test compounds were used earlier to study the VUV APPI ionization process,^4^ allowing comparison of the soft X-ray APPI with the VUV-APPI. In the LC-MS applications, the ionization process using soft X-ray APPI was initiated by the ionization of the solvent molecules used as eluents in the LC and gas molecules (nebulizing gas and atmospheric gases) present in the ionization zone. Here, the energy of the soft X-ray photons was 4.9 keV, which is sufficient to release valence electrons and possibly also inner shell electrons from the eluents and atmospheric gases. The primary electrons formed in the ionization process were rapidly thermalized close to 0 eV and could be captured by the molecules having positive EAs. Since oxygen (EA = 0.451 eV)^17^ exists in the soft X-ray APPI source in much higher concentrations than the analyte molecules, it is evident that oxygen is first ionized to superoxide ions (O_2_^–•^), similar to the situation in APCI^18^ and VUV APPI.^4^

In the gas phase, O_2_^–•^ is a relatively strong base (see below) and can react directly with an analyte (M) by a proton transfer reaction, producing deprotonated molecules ([M – H]^−^). In addition, O_2_^–•^ can initiate the formation of deprotonated solvent molecules, which can in turn deprotonate an analyte if the gas-phase acidity of the analyte exceeds the acidity of the solvent molecule, i.e., if the Δ*G*_acid_ (M) is lower than the Δ*G*_acid_ (solvent). The soft X-ray APPI mass spectra of 2-naphthol and 2-naphthoic acid showed intense deprotonated molecules (Figure 1). The gas-phase acidities of 2-naphthol and 2-naphthoic acid are about 1408 kJ mol^–1^^19^ and 1370 kJ mol^–1^ (estimated),^4^ respectively. Since the gas-phase acidity of HO_2_^•^ (1451 kJ mol^–1^)^17^ is higher than those of 2-naphthol and 2-naphthoic acid, proton transfer reactions with O_2_^–•^ or deprotonated solvent molecules can occur, allowing formation of deprotonated molecules of 2-naphthol and 2-naphthoic acid.

Charge-exchange reactions in the negative-ion mode are possible if an analyte has higher EA than that of a reactant molecule. The EAs of 1,4-naphthoquinone (1.813 eV), 1,4-dinitrobenzene (2.003 eV), and hexachlorobenzene (0.915 eV) are higher than that of O_2_ (0.451 eV),^17^ and these compounds are ionized by a charge exchange reaction with O_2_^–•^, producing a negative molecular ion (M^–•^). Deprotonated molecules were not detected, because these compounds do not include an acidic group and cannot react by a proton transfer reaction.

The soft X-ray APPI mass spectra also showed substitution ions [M – X + O]^−^ formed by oxidation reactions with O_2_^–•^ or with other reactive oxidation species^20^ possibly formed in the soft X-ray APPI process. The spectra of 1,4-naphthoquinone and 2-naphthol showed [M – H + O]^−^ ions at *m/*z 173 and *m/*z 159, respectively, whereas the spectra of hexachlorobenzene showed abundant [M – Cl + O]^−^ ion at *m*/*z* 263 with a characteristic chlorine isotope pattern. Some fragment ions were also detected. The spectra of 2-naphthoic acid showed ion [M – H – CO_2_]^−^ at *m*/*z* 127, and the spectra of 1,4-dinitrobenzene showed ion [M – 30]^−^ at *m*/*z* 138, which is formed either by the loss of NO or by a substitution reaction producing ion [M – NO_2_ + O]^−^.

**Figure 2:**
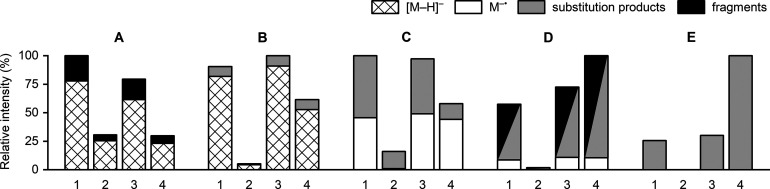
Comparison of the ionization efficiencies of soft X-ray and VUV APPI with and without dopant (toluene) in the negative ion mode. The comparison measurements were done using infusion of the mixture of compounds in acetonitrile/water (50/50). The bars represent absolute abundances of the total ion currents and proportions of [M – H]^−^, M^–•^, substitution products, and fragments. A = 2-naphthoic acid, B = 2-naphthol, C = 1,4-naphthoquinone, D = 1,4-dinitrobenzene, and E = hexachlorobenzene. 1 = soft X-ray APPI without dopant, 2 = VUV APPI without dopant, 3 = soft X-ray APPI with a dopant, 4 = VUV APPI with a dopant.

All the spectra of the test compounds measured by dopant-assisted VUV APPI with 10 eV photons are very similar to the spectra measured with soft X-ray APPI with 4.9 keV photons. This suggests that the reactant ion composition is similar in negative ion soft X-ray and VUV APPI. However, the advantage of soft X-ray APPI is that no dopant is needed to achieve high ionization efficiency in the negative ion mode, as shown in Figure 2, which presents a comparison of the ionization efficiencies between soft X-ray and VUV APPI with and without use of a dopant (toluene). The results show that the ionization efficiency is about 10–50 times better with soft X-ray than with VUV APPI without the dopant. However, the use of dopant in VUV APPI significantly increased ionization efficiency achieving a level similar to that of soft X-ray APPI without dopant. In contrast, addition of the dopant did not significantly affect the ionization efficiency in the soft X-ray APPI. These results indicate that the formation of electrons directly from commonly used LC eluents, such as acetonitrile, methanol, and water, or atmospheric gases, is not sufficient to achieve maximum sensitivity with 10 eV VUV photoionization. This is because the IEs of the LC eluents or atmospheric gases are higher than 10 eV. However, the addition of a dopant having IE below 10 eV can efficiently produce thermal electrons in VUV photoionization, which explains the significant improvement in ionization efficiency with dopant-assisted VUV APPI in comparison to VUV APPI without the use of a dopant. The energy of the soft X-ray photons was 4.9 keV, high enough to efficiently release valence electrons and often also inner shell electrons without the use of a dopant from all kinds of molecules, including LC eluents and atmospheric gases, resulting in high numbers of thermal electrons and efficient ionization in the negative ion mode. For the same reason, the addition of dopant did not improve the ionization efficiency in soft X-ray APPI. The high ionization efficiency in negative ion soft X-ray APPI is clearly an advantage in comparison to dopant-assisted VUV APPI, because the use of a dopant complicates the analytical system and commonly used dopants (such as toluene and chlorobenzene) are harmful to the environment and health.

**Figure 3:**
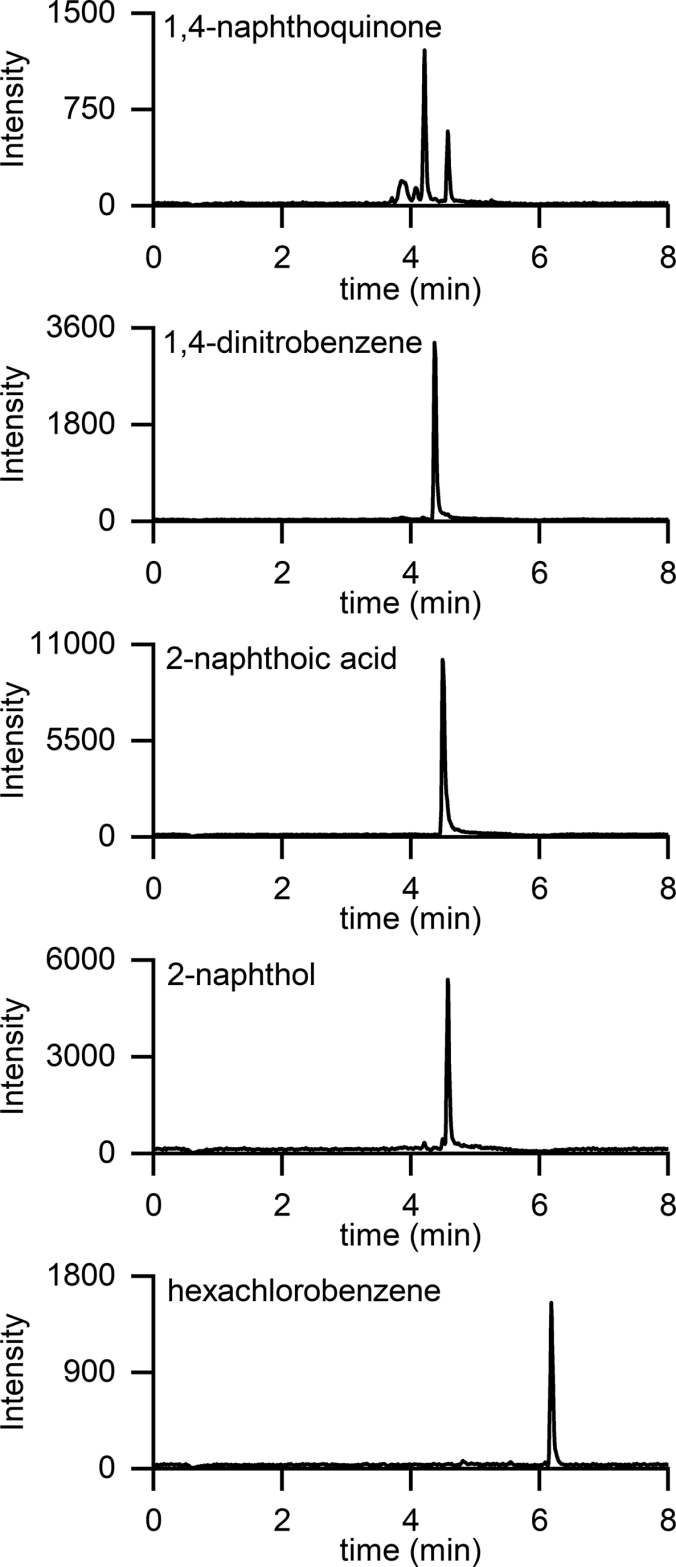
Selected ion chromatograms of the test compounds measured by LC-MS using soft X-ray APPI without a dopant. The selected ions were the following: *m*/*z* 158 and 173 for 1,4-naphthoquinone, *m*/*z* 168 and 138 for 1,4-dinitrobenzene, *m*/*z* 171 and 127 for 2-naphthoic acid, *m*/*z* 159 and 143 for 2-naphthol, and *m*/*z* 263, 265, 267, and 269 for hexachlorobenzene.

We also demonstrated the use of soft X-ray APPI in analysis of the test compounds by LC-MS. The flow rate was 0.4 mL min^–1^, the gradient consisted of acetonitrile and water, and no dopant was used. The soft X-ray APPI LC-MS chromatograms presented in Figure 3 are the sum of the ion currents of the ions detected (*m*/*z* 158 and 173 for 1,4-naphthoquinone, *m*/*z* 168 and 138 for 1,4-dinitrobenzene, *m*/*z* 171 and 127 for 2-naphthoic acid, *m*/*z* 159 and 143 for 2-naphthol, and *m*/*z* 263, 265, 267, and 269 for hexachlorobenzene). The five peaks, in order of elution, correspond to the signals for 1,4-naphthoquinone (4.2 min), 1,4-dinitrobenzene (4.4 min), 2-naphthoic acid (4.5 min), 2-naphthol (4.6 min), and hexachlorobenzene (6.2 min) with about 200 pmol injected into the column. All the test compounds were readily detected in the ion chromatograms. The repeatability was tested with six LC-MS runs. The relative standard deviation was 5.2% for 1,4-naphtoquinone, 5.5% for 1,4-dinitrobenzene, 1.1% for 2-naphthoic acid, 3.6% for 2-naphthol, and 3.3% for hexachlorobenzene, indicating good repeatability of the LC-MS method using soft X-ray ionization.

The limits of detection currently furnish no meaningful information because the soft X-ray setup was an early prototype and the ionization conditions as well as the LC method were not fully optimized. However, comparison between soft X-ray APPI without a dopant and dopant-assisted VUV APPI suggests that soft X-ray APPI can reach at least similar sensitivity as VUV APPI in the negative ion mode.
